# Parasitology should not be abandoned: data from outpatient parasitological testing in Guangdong, China

**DOI:** 10.1186/s40249-017-0332-0

**Published:** 2017-09-04

**Authors:** Lan-Gui Song, Xiao-Ying Zheng, Da-Tao Lin, Guang-Xi Wang, Zhong-Dao Wu

**Affiliations:** 1 Department of Parasitology of Zhongshan School of Medicine, Sun Yat-sen University, Guangzhou, 510080 China; 2 Key Laboratory of Tropical Disease Control (SYSU), Ministry of Education, Guangzhou, 510080 China; 3 Provincial Engineering Technology Research Center for Biological Vector Control, Guangzhou, Guangdong 510080 China; 4 Southwest Medical University, Luzhou, Sichuan 646000 China

**Keywords:** Parasitic diseases, Parasitology, Guangdong Province, P. R. China

## Abstract

Over the past six decades, the Chinese government made parasitoses with a high disease burden, including soil-transmitted nematode infections, malaria, leishmaniasis, filariasis, and schistosomiasis, a public health priority because they were seen to be crucial impediments to the development of rural areas. As a result, these debilitating parasitic diseases that used to be widely prevalent have been well controlled or eliminated. Consequently, less attention has been paid to parasitic infection during the rapid development of the economy, especially in developed areas. However, our investigations conducted in the parasitological laboratory of Sun Yat-sen University (Guangzhou, Guangdong, China) show that emerging parasitic diseases still threaten many people’s health, with 340 of 880 outpatients (38.6%) receiving a diagnosis of parasitic disease, among whom 201 (59.1%) had clonorchiasis and 120 (35.3%) had taeniasis/cysticercosis. Furthermore, our doctors are not equipped with sufficient parasitology knowledge because this discipline is not able to maintain attraction. Many parasitic infections that result in severe consequences are treatable and preventable, but the phenomena of misdiagnosis and missed diagnosis are common and merit attention.

**Electronic supplementary material:**

The online version of this article (doi:10.1186/s40249-017-0332-0) contains supplementary material, which is available to authorized users.

## Multilingual abstracts

Please see Additional file 1 for translations of the abstract into the five official working languages of the United Nations.

## Background

China used to suffer greatly from parasitic infections [1, 2]. For example, lymphatic filariasis was highly prevalent in China 60 years ago, with 31 million cases and 330 million people at risk of endemic infection living in 864 counties of 16 provinces, autonomous regions, and municipalities. Its most spectacular symptom is elephantiasis — edema with thickening of the skin and underlying tissues that causes dramatic loss of labour to society, followed by tremendous economic loss [3, 4]. To enhance the population’s health, China spared no effort to fight against parasites over the past six decades, which led to great progress in parasitic disease control. For example, the conventional major parasitic diseases in China (soil-transmitted nematode infections, malaria, leishmaniasis, filariasis, and schistosomiasis) either have been eliminated or are well controlled [3–9]. Therefore, less attention is paid to parasite control than before. Instead, non-communicable chronic diseases such as cancer, diabetes, respiratory diseases, hypertension, and dementia are becoming common causes of disability and mortality [10, 11], and therefore, studies in this field are increasingly popular, and parasitology is no longer a hot discipline. Parasitology used to be a compulsory and key course for all medical students in China, but this is no longer the case. More and more medical schools have decided to devote fewer resources to the course. Hence, the scale of parasitology becomes smaller and smaller [12, 13]. As a result, departments of parasitology in China have been abrogated or integrated to pathogenic biology or pathogen and immunology [14]. Furthermore, the time spent studying parasitology has been significantly shortened in some medical schools in China [12, 15].

Parasitic infections are becoming neglected diseases, especially in well-developed areas with good sanitary and hygienic conditions, such as Shanghai, Beijing, and Guangdong Province in mainland China, because parasitoses are generally recognized as diseases of poverty [16] that will disappear as a result of higher standards of living and poverty alleviation. However, as a result of an unhealthy diet and lifestyle, an increasing number of people are willing to eat raw or undercooked vegetables and meat (pork, beef, fish, crab, snails, etc.), especially in highly developed cities like Guangzhou and Shenzhen in Guangdong. Thus, the prevalence of foodborne parasitic diseases is actually on a slight rise [17–20]. In addition, many urban families are keeping pets like cats (a known host of *Toxoplasma gondii*), which increases the risk of infection [21]. Furthermore, as national and international travel become more and more accessible and affordable, adventurous travellers will continue to return home with increasingly exotic infections, including parasitic infections [22]. This study aimed to investigate the parasitic infection situation in Guangdong, China, and to call attention to these ancient infectious diseases, especially in highly developed areas.

## Patients and methods

**Fig. 1:**
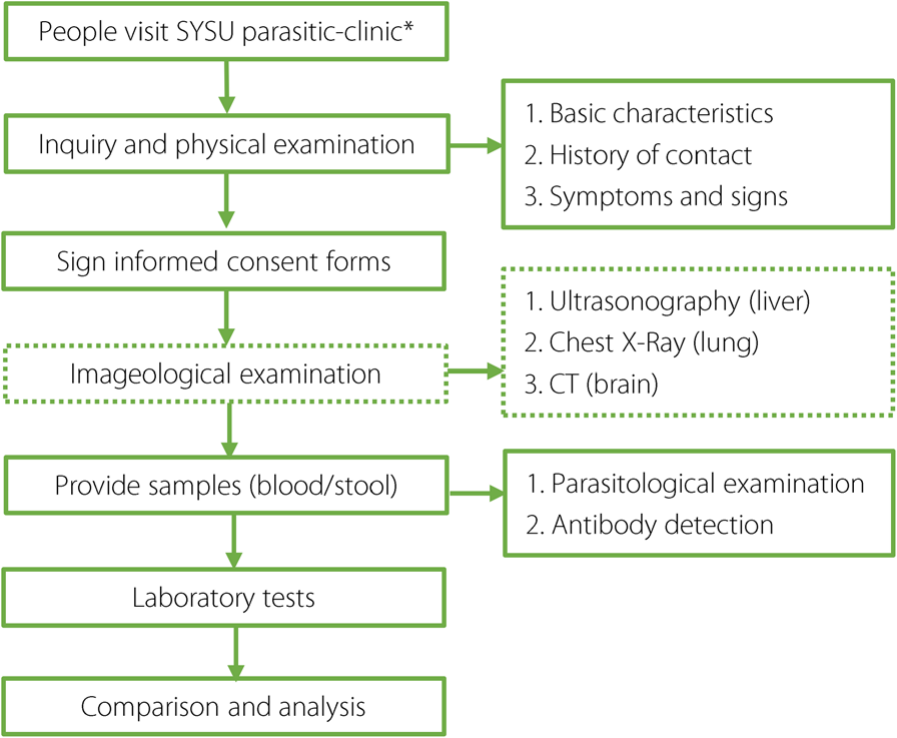
Flowchart. *Full line*: All participants took these steps. *Dotted line*: Not all participants took this step. ^*^ *SYSU parasitic-clinic*: Parasitological Laboratory of Sun Yat-sen University

The flowchart of this study is shown in Fig. 1. Between 1 Sept 2015 and 31 Aug 2016, blood and/or stool samples from 880 suspected cases were tested in the parasitological laboratory of Sun Yat-sen University (SYSU parasitic-clinic). The SYSU parasitic-clinic has gained a good reputation in the diagnosis of parasitic diseases, and experienced doctors generally refer suspected patients to the SYSU parasitic-clinic because their hospitals are not equipped with appropriate tests and skilled technicians. Each of the suspected patients was interviewed by a well-trained staff member. The information collected included the following.

1. Demographic characteristics: gender, age, education level, household income, and place of residence (i.e., city and province);
2. History of contact: drinking unclean water, consumption of raw or undercooked vegetables or meat (pork, beef, fish, crab, snails, etc.), keeping pets or contact with pets, contact with cecariae-infested water;
3. Symptoms and signs: stomachache, diarrhoea, jaundice, cough, expectoration, subcutaneous nodule, epilepsy, paralysis, impaired vision, or loss of vision.

Clinical diagnosis was made based on contact history, symptoms and signs, and laboratory findings. Imaging studies also helped in diagnosis, but not all suspected participants underwent such studies for many reasons, such as high cost and fear of radiation.

### Parasitological examination

For patients in whom intestinal parasitic diseases were suspected, a single faecal sample was collected in a capped plastic polypot. Triplicate Kato-Katz microscope slides were then prepared and examined under a microscope at × 400 magnification by two experienced technicians within 2 h [23].

### Antibody tests

Venous blood samples were drawn from each participant in aseptic conditions without stasis using vacuum blood collection tubes (no additive). Blood serum was obtained after rapid centrifugation for 10 min at 2000 *g* and then stored in 0.5 ml aliquots at −20 °C. The specific antibody for detection was chosen according to doctors’ advice based on the participant’s contact history and manifestations, and detection was then carried out by two skilled technicians in strict accordance with the manufacturers’ instructions. The antibody test kits were purchased from Yikang Co. Ltd., Guangzhou, China.

### Statistical analysis

For continuous variables, data were expressed as mean (*M*) ± standard deviation (*SD*), and differences between groups were examined with Student’s *t*-test; for categorical variables, data were expressed as number (%), and differences between groups were examined with the chi-square or Fisher’s exact test. A *P* value of less than 0.05 (two-sided) was considered to indicate statistical significance. All data analyses were performed with SPSS 22.0 software (Chicago, IL).

## Results and discussion

A significant difference in gender was seen between infected and non-infected participants, but little difference was observed in age, education level, household income, and place of residence (Table 1), which implies that these factors do not have significant effects on the morbidity of parasitic diseases. Women acquired fewer parasitic infections, partially due to better hygiene-related behaviours, because woman usually pay more attention to their own health condition.

**Table 1:** Comparison of characteristics between infected and non-infected participants

Factors	Infected	Non-infected	*P* value
Gender (%)			
Male	258 (75.9)	265 (49.1)	0.001
Female	82 (24.1)	275 (50.9)	
Age (y)	37.6 ± 14.4	42.4 ± 14.2	0.146
Education Level (%)			
Illiterate	0 (0.0)	15 (2.8)	0.449
Primary school	13 (3.8)	36 (6.6)	
Middle school	128 (37.7)	193 (35.8)	
College or above	199 (58.5)	296 (54.8)	
Household income (%)			
< 10,000 RMB	188 (55.2)	246 (45.5)	0.482
10,000 to 50,000 RMB	45 (13.2)	104 (19.3)	
50,000 to 100,000 RMB	18 (5.3)	67 (12.4)	
> 100,000 RMB	89 (26.3)	123 (22.8)	
Place of residence (%)			
Urban areas	331 (97.4)	506 (93.7)	0.014
Rural areas	9 (2.6)	34 (6.3)	

**Fig. 2:**
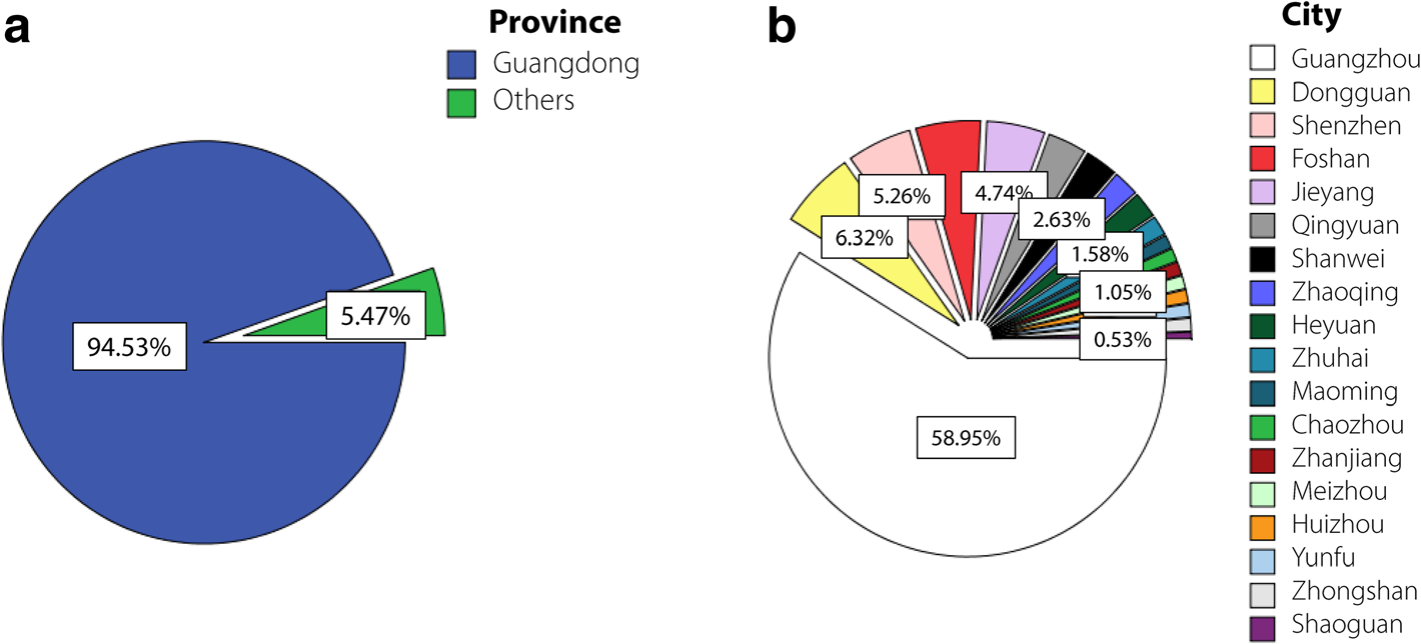
Geographic distribution (provinces, cities) of the 880 suspected cases in SYSU parasitic-clinic. **a** Distribution by province. **b** Distribution by city in Guangdong Province (picture drawn with SPSS 22)

**Fig. 3:**
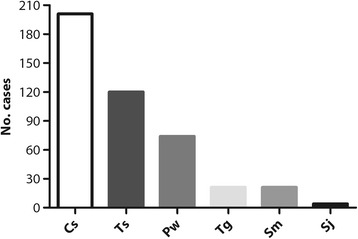
Number of clinically confirmed cases of the six parasitic diseases in SYSU parasitic-clinic. Cs: *Clonorchis sinensis*; Pw: *Paragonimus westermani*; Tg: *Toxoplasma gondii*; Ts: *Taenia solium*, Sm: *Spirometra mansoni*; Sj: *Schistosoma japonica* (picture drawn with GraphPad Prism 5)

**Fig. 4:**
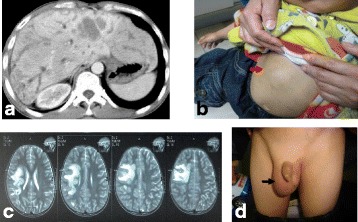
Damage caused by the main two parasitic infections. **a** CT image of intrahepatic biliary dilatations caused by *Clonorchis sinensis* infection. **b** MRI of brain cyst due to cerebral paragonimiasis. **c** Patient presented with a subcutaneous nodule in his right upper abdomen (*red arrow*) due to cutaneous paragonimiasis. **d** A patient presented with a mass in his right scrotum (*black arrow*) due to cutaneous paragonimiasis. All pictures are reproduced with the permission of the patient or that of his or her guardians

As seen in Fig. 2, more than 94% of cases were from Guangdong; among these cases, around 60% were from Guangzhou, the provincial capital of Guangdong, and the others were from other cities, including Shenzhen, Dongguan, and Foshan. This distribution is understandable because the SYSU parasitic-clinic is located in Guangzhou and primarily provides health services to the local population. Of the 880 suspected cases, 340 participants were confirmed to have at least one of the six parasitic diseases (Fig. 3), and some even had co-infections. The leading parasitic disease was clonorchiasis, probably as a result of local eating habits. Many patients enjoy sashimi (raw fish), and this habit is difficult to change, even after long-term health education [19, 24]. However, many cases of clonorchiasis are asymptomatic or have mild symptoms, and the infection may go unrecognized or be misdiagnosed. Recent studies have shown that it is capable of causing cancer of the liver and bile duct, and in 2009 it was classified as a group 1 biological carcinogen that poses a remarkable threat to human health [25]. Taeniasis/cysticercosis, usually caused by ingestion of raw pork, is second only to clonorchiasis. Patients may show severe symptoms, such as epilepsy, paralysis, and impaired vision or loss of vision, leading to reduced physical functioning [2]. As a debilitating illness, paragonimiasis takes third place, manifesting as cough, bloody sputum, and chest pain [17] (Fig. 4).

**Fig. 5:**
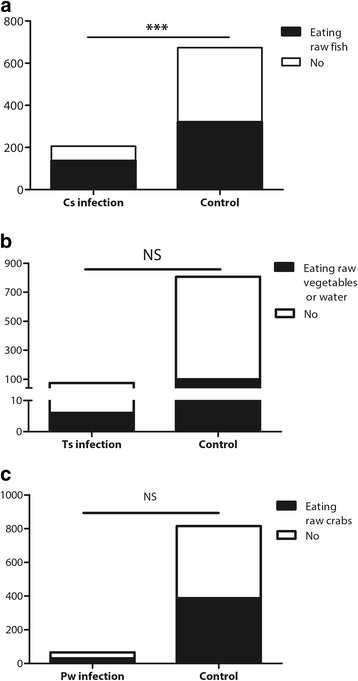
History contact frequencies between infected and non-infected participants. **a** Eating raw or undercooked fish. **b** Eating raw or undercooked crab or crayfish. **c** Eating raw or undercooked pork or vegetables or drinking unclean water. Cs: *Clonorchis sinensis*. Ts: *Taenia solium*. Pw: *Paragonimus westermani*. ***: *P* < 0.001. NS: not significant. (This picture was drawn with GraphPad Prism 5)

As shown in Fig. 5, patients with clonorchiasis had a much higher incidence of raw fish consumption than other participants (*χ* ^2^ = 22.53, *P* < 0.001), which means that ingestion of sashimi is a common risk factor that contributes to an accurate diagnosis. However, few patients with cysticercosis or paragonimiasis reported a contact history, which hindered an accurate diagnosis. As a result, clinical misdiagnosis and missed diagnosis often occurs.

As ancient pathogens, human parasites have long been combated [2]. Because parasitic diseases are treatable and preventable, unremitting efforts have led to the control or elimination of the five main parasitic diseases in China [3–9]. Nationally, the annual incidences of schistosomiasis (0.2133/100000), malaria (0.2326/100000), filariasis (0), and leishmaniasis (0.0223/100000) are now very low according to the available 2016 official record [26]. Significant improvement has also been observed in the control of soil-transmitted nematode infection; the prevalence dropped from 20.88% in 2006 to 3.12% in 2013 [27]. However, other parasitic diseases that are less prevalent and relatively less harmful to humans have attracted little attention from the government. As a result of an unhealthy diet and lifestyle, an increasing number of people are willing to eat raw or undercooked vegetables and meat (pork, beef, fish, crab, snails, etc.), especially in highly developed cities like Guangzhou and Shenzhen. Hence, the prevalence of foodborne parasitic diseases is actually on a slight rise. The occurrence of emerging parasitic diseases is occasionally reported and has at times raised huge concern [1, 18, 28, 29]. For instance, an outbreak of eosinophilic angiostrongylaisis caused by the consumption of raw *Ampullaria gigas* in Beijing in 2006 caused panic, and at that time, many residents refused to eat freshwater snails (the intermediate host of *Angiostrongylus cantonensis*) for fear of acquiring infection [18]. However, this fear was soon forgotten.

Time has devalued the stature of parasitology, which seems unavoidable. Misdiagnosis and missed diagnosis of parasitic diseases are often seen in current practice [30–33], partially due to the lack of availability of deworming tablets and local expertise, especially in non-endemic settings [22]. For example, it was estimated that more than 6 million people in Guangdong Province had clonorchiasis, but only 1.8 million were treated, indicating a large number of missed diagnoses and misdiagnosed cases due to the non-specific symptoms and the lack of proper training of physicians [34, 35]. Hepatic clonorchiasis might be misdiagnosed as hepatitis B infection because of the geographic overlap and similar clinical features of these two diseases. Hence, diagnosis and treatment of clonorchiasis in a case of co-infection with hepatitis B virus is a great challenge [29, 36]. Those that parasitize in the human brain, such as juvenile *Spirometra mansoni* and *Taenia solium*, can lead to severe brain damage or death without timely anthelmintic treatment [2, 32, 37]; these are easily treatable diseases, but neurologists usually consider brain tumours instead of parasitic infections. In addition, it is quite difficult to differentiate from other diseases under the circumstance of ectopic parasitism. Thus, it is vital to equip doctors with knowledge of parasitology so that they are qualified to make an accurate diagnosis so that patients can receive timely treatment.

This study has some limitations. We only studied one-year data from one small clinic, even though it is the largest clinic of parasitology in Guangdong. However, it is only the tip of the iceberg and cannot represent the whole picture of the parasitic disease burden there. A great number of cases of parasitic diseases go undiagnosed because medical students’ or doctors’ knowledge of parasitology is far from satisfactory. Guangdong has the largest economy in China, and its gross domestic product has ranked 1st for 27 consecutive years, at over seven trillion RMB (around 1.1 trillion USD) in 2015 [38, 39]. Guangdong is one of the most developed areas in China, but parasitic diseases, usually recognized as a disease of poverty, remain a problem and pose a threat to human health. It is important to raise the awareness of both the public and medical personnel about parasitic diseases. Not only would it help to reduce the prevalence of parasitoses, but it would also decrease the rates of missed diagnosis or misdiagnosis.

## Conclusions

Parasitic diseases still threaten many people’s health, not only in rural areas but also in urban areas. However, our doctors are not equipped with sufficient knowledge in parasitology because this discipline has not been able to maintain its attraction. Many parasitic infections that result in severe consequences are treatable and preventable, but the phenomena of misdiagnosis and missed diagnosis are common, which merits attention.

## Additional file

Additional file 1: Multilingual abstracts in the five official working languages of the United Nations. (PDF 530 kb)
