# Supplementary material for: Parasitology should not be abandoned: data from outpatient parasitological testing in Guangdong, China
Source: Infect Dis Poverty. 2017 Sep 4;6:119. doi: 10.1186/s40249-017-0332-0 (PMC5582392; doi:10.1186/s40249-017-0332-0)
Supplement: Additional file 1: — Multilingual abstracts in the five official working languages of the United Nations. (PDF 530 kb) [file 40249_2017_332_MOESM1_ESM.pdf]

Translation of the abstract into the five official working languages of the United Nations

لا ينبغي التخلي عن علم الطفيليات: بيانات من الفحص الطفيلي للمرضى الخارجيين في جواندون، الصين

Lan-Gui Song, Xiao-Ying Zheng, Da-Tao Lin, Guang-Xi Wang and Zhong-Dao Wu

المستخلص

على مدى العقود الستة الماضية، جعلت الحكومة الصينية الطفيليات عبئا مرضياً كبيراً، بما في ذلك عدوى الديدان الخيطية التي تنتقل عن طريق التربة، والملاريا، وداء الليشمانيا، وداء الفيلاريا، وداء البلهارسيا، وهي أولوية للصحة العامة لأنها اعتبرت عقبات حاسمة أمام تنمية المناطق الريفية. ونتيجة لذلك، فإن هذه الأمراض الطفيلية المنهكة التي كانت منتشرة على نطاق واسع قد تمت السيطرة عليها أو القضاء عليها بشكل جيد. وبالتالي، تم إيلاء اهتمام أقل للعدوى الطفيلية خلال التطور السريع للاقتصاد، وخاصة في المناطق المتقدمة. غير أن التحقيقات التي أجريناها في المختبر الطفيلي بجامعة صن يات صن (جوانجو، جواندون، الصين) تبين أن الأمراض الطفيلية الناشئة لا تزال تهدد صحة العديد من الناس، حيث يتلقى 340 من 880 مريضا خارجيا (38.6٪) تشخيصاً للأمراض الطفيلية، منهم 201 (59.1٪) لديهم داء كلونورشياسيس (clonorchiasis) و 120 (35.3٪) لديهم تينياسيس / سيستيسركوسيس (taeniasis/cysticercosis). علاوة على ذلك، فإن أطبائنا ليسو مجهزين بالمعرفة الكافية للطفيليات لأن هذا الانضباط ليس له القدرة بالحفاظ على جاذبيتها. العديد من العدوى الطفيلية التي تؤدي إلى عواقب وخيمة قابلة للعلاج ويمكن الوقاية منها، ولكن ظواهر التشخيص الخاطئ والتشخيص الفائت شائعة وتستحق الاهتمام.

Translated from English version into Arabic by Khaled Zayed

中国广东门诊患者的寄生虫学检测数据表明仍需重视寄生虫学

Langui Song, Xiaoying Zheng, Datao Lin, Guangxi Wang, Zhongdao Wu

摘要：
在过去的六十余年，土源性线虫病、疟疾、利什曼原虫病和血吸虫病等寄生虫病引起了较高的疾病负担，严重阻碍农村地区的发展，因此中国政府把这些寄生虫病作为公共卫生优先事项。过去广泛流行的这些寄生虫病目前已经被良好控制甚至消除。在经济快速发展时期，人们较少关注寄生虫感染问题，特别是在发达地区。然而，我们在这中国广东省广州中山大学寄生虫学实验室的调查研究发现，新发寄生虫病仍旧威胁人类健康，在 880 位门诊患者中，诊断出 340 例患者被寄生虫感染（38.6%：340/880），其中 201（59.1%）例为华支睾吸虫病、120（35.3%）例为猪带绦虫病/囊虫病。此外，由于寄生虫学科发展受限，本院医生不具备充足的寄生虫学知识。尽管许多会导致严重后果的寄生虫病是可防可治的，但是误诊与漏诊的现象仍十分常见，值得关注。

Translated from English version into Chinese by Lan-Gui Song

Caractère indispensable de la parasitologie: données de tests parasitologiques chez des patients non hospitalisés à Guangdong, Chine

Lan-Gui Song, Xiao-Ying Zheng, Da-Tao Lin, Guang-Xi Wang and Zhong-Dao Wu

Résumé
Au cours des six dernières décennies, le gouvernement chinois a fait des parasitoses à fort taux de morbidité (les géohelminthiases, le paludisme, la leishmaniose, la filariose et la schistosomiase) une priorité de santé publique parce qu'elles étaient considérées comme des obstacles majeurs au développment des zones rurales. De ce fait, ces maladies parasitaires débilitantes autrefois largement répandues ont été bien contrôlées ou éliminées. Par conséquent, avec le développement rapide de l'économie, peu d'attention est accordée aux infections parasitaires, en particulier dans les régions développées. Toutefois, nos recherches menées dans le laboratoire de parasitologie de l'Université Sun Yat-sen (Guangzhou, Guangdong, Chine) montrent que les maladies parasitaires continuent de menacer la santé de nombreuses personnes avec 340 sur 880 (38,6%) patients non

hospitalisés ayant reçu un diagnostic de maladie parasitaire, dont 201 (59,1%) avaient la clonorchiase et 120 (35,3%) la taeniase/cysticercose. En outre, nos médecins ne sont pas suffisamment équipés de connaissances parasitologiques parce que cette discipline devient peu attrayante. Nombreuses infections parasitaires entraînant des conséquences graves sont traitables et évitables, mais l'ampleur des mauvais diagnostics ou des diagnostics ratés devient courante et mérite d'attirer l'attention.

Translated from English version into French by Kokouvi Kassegne

Паразитология не следует отказываться: данные из паразитологических испытаний амбулаторных пациентов в провинции Гуандун, Китай

Lan-Gui Song, Xiao-Ying Zheng, Da-Tao Lin, Guang-Xi Wang and Zhong-Dao Wu

Введение
За последние шесть десятилетий, китайское правительство признaлось, что паразитозов с высоким бременем болезни, в том числе и нематодные инфекции, передаваемые через почву, малярия, лейшманиоз, филяриоз и шистосомоз является приоритетом общественного здравоохранения, поскольку они рассматриваются как важнейшие препятствия на пути развития сельских территорий. В результате этих изнурительных паразитарных заболеваний, которые раньше были широко распространены и были хорошо контролируемы или исключены. Следовательно, меньше внимания было уделено паразитарным инфекциям во время быстрого развития экономики, особенно в развитых районах. Однако, наши исследования, проведенные в паразитологической лаборатории университета Сунь ятсена (Гуанчжоу, Гуандун, Китай) показывались, что возникающие паразитарные болезни по-прежнему угрожают здоровью многих людей, 340 из 880 амбулаторных пациентов (38.6%) получили диагноз паразитарного заболевания, среди которых 201 (59,1%) имело клонорхоза и 120 (35.3%) имели тениоз/цистицеркоз. Кроме того, наши врачи не оборудованы достаточным знаниями по паразитологии, потому что эта дисциплина не в состоянии поддерживать привлекательность. Много паразитарных инфекций, которые приводят к серьезным последствиям, не поддающихся лечению и профилактике, но явления ошибочного диагноза и пропущенные диагноыз распространены и заслуживают внимания.

Translated from English version into French by Hao-Qi Zhang

La parasitología no debe ser abandonada: datos de pruebas parasitológicas ambulatorias en Guangdong, China

Lan-Gui Song, Xiao-Ying Zheng, Da-Tao Lin, Guang-Xi Wang and Zhong-Dao Wu

Abstracto
Durante las últimas seis décadas, el gobierno de China hizo de las parasitosis con de alta incidencia, incluyendo las infecciones por nematodos transmitidas por el suelo, la malaria, la leishmaniasis, la filariasis y la esquistosomiasis, una prioridad de salud pública porque eran impedimentos cruciales para el desarrollo de áreas rurales. Como resultado, estas enfermedades parasitarias debilitantes que solían ser ampliamente prevalentes han sido bien controladas o eliminadas. Por consiguiente, se ha prestado menos atención a la infecciones parasitarias durante el rápido desarrollo de la economía, especialmente en las zonas desarrolladas. Sin embargo, nuestras investigaciones realizadas en el laboratorio parasitológico de la Universidad Sun Yat-sen (Guangzhou, Guangdong, China) muestran que las enfermedades parasitarias emergentes siguen amenazando la salud de muchas personas, con 340 de 880 pacientes ambulatorios (38.6%) recibiendo un diagnóstico de enfermedad parasitaria, entre los cuales 201 (59.1%) tuvieron clonorquiasis y 120 (35.3%) taeniasis / cisticercosis. Además, nuestros médicos no están equipados con suficientes conocimientos de parasitología porque esta disciplina no es capaz de mantener la atracción. Muchas infecciones

parasitarias que resultan en consecuencias graves son tratables y prevenibles, pero los fenómenos de diagnóstico erróneo y falta de diagnóstico son comunes y merecen atención.

Translated from English version into French by Laura C Vicente Rodriguez
